# Psychometric properties of the Sport Courage Scale for Chinese athletes

**DOI:** 10.3389/fpsyg.2023.1133720

**Published:** 2023-06-07

**Authors:** Shenmao Gao, Zeyou Guo, Renfang Zhang, Junze Jin, Guangbo Dou

**Affiliations:** College of Kinesiology, Shenyang Sport University, Shenyang, China

**Keywords:** Chinese athletes revision, courage, sport courage, SCS, characteristics of psychometrics, exploratory structural equation modeling

## Abstract

**Objective:**

To revise *Sport Courage Scale* (SCS) suitable for Chinese athletes.

**Methods:**

Six hundred and eighty three athletes were selected for verification factor analysis, correlation analysis, reliability analysis, and independent sample *t*-test using the method of random sampling of the entire group.

**Results:**

Confirmatory factor analysis model showed that model 1 (25 items) failed to fit the data; while model 2 (20 items) was finally accepted with its five-factor model. The factor structure consists of 5 dimensions (χ^2^/df = 2.262;CFI = 0.969;TLI = 0.963; RMSEA = 0.043; SRMR = 0.044). Cronbach’s *α* of the final version of *SCS* was 0.845, and the corrected correlation coefficient between the items and the total score of the scale was between 0.352 and 0.788.

**Conclusion:**

Revised *SCS* has good reliability and validity and can be used as a measurement tool for the sports courage of athletes in China.

## Introduction

Courage is a voluntary will to act against threats with varying degrees of fear/without fear to achieve an important moral goal or aim (Woodard and Pury, 2007). Putman (1997) proposed three types of courage, namely physical courage, moral courage, and psychological courage. Physical courage is defined as the action taken in the face of physical danger; moral courage defined as the truth and integrity in the face of danger; psychological courage defined as the action taken regardless of the risks to one’s mental health (Putman, 1997). Corlett (1996) believed that courage can be used as a tool to manage and overcome fear, anxiety, tension, and stress in sports. Courage may be related to various popular topics in sports psychology, such as self-confidence, concentration, achievement motivation, competitiveness, psychological resilience, decisiveness, and response to improve motor function and performance (Konter, 2013). As found in the study of Konter et al. (2020a), football players with higher overall courage levels have more sports experience in football. When it comes to courage, experts and scholars in sports have shown the importance of courage to success and operational performance. However, sports courage and related concepts have received little attention (Konter, 2013).

Athletes are a relatively special group and may have high anxiety and depression in different periods, which directly affects athletes’ mental state and training. Ping and Qiu (2016) found that people who put themselves in a frightening stimulus or scene to participate in brave behavior have a lower probability of suffering from serious anxiety problems. Mann et al. (2007) found that the three most common non-injury-related topics discussed in the survey of athletes are stress/pressure, anxiety, and burnout. Courage has an important positive influence on athletes’ personality cultivation and coping in daily life (Konter and Ng, 2012). Sports are an essential part of athletes’ daily life, so it is necessary to further study athletes’ sports courage.

There are many tools for courage measurement. The most common ones are *Personal Perspectives Survey* (Woodard, 2004), *Woodard Pury Courage Scale* (Woodard and Pury, 2007), *Values in Action Inventory of Strengths* (Peterson and Seligman, 2004), and *Courage Measur*e (Norton and Weiss, 2009). All the above measure multidimensional courage from different angles, while *SCS* (Konter and Ng, 2012) measures courage in sports. Upon preparation of PPS questionnaires, the subjects are selected from several biased samples with high fear and high willingness to act, and there are items with multiple loads. The overall questionnaire shows good Cronbach’s *α*; however, there may be moral judgment in the courage measurement, which may lead to measurement deviations (Qian et al., 2016).

The controversy with WPCS is that the items in the scale are more related to morality and occupation, and courage is not evaluated as a whole. Moreover, WPSC Cronbach’s *α* of WPSC is 0.683, with three double-load items. As a result, the reliability and validity index of WPCS is not ideal (Qian et al., 2016). Compared with PPS-31, WPCS questions and reliability have been reduced. Besides, questions such as the empirical validity of the questionnaire have yet to be verified (Cheng and Huang, 2014). *VIA*-IS scale is developed under the background of an American individualist culture, so measuring courage with 4 items, including courage, persistence, integrity, and vitality, may not apply to countries with different cultures, especially to Chinese people who have lived in collectivist culture for a long time. The applicability of *VIA*-IS may decline (Cheng and Huang, 2014). The operational definition used in the development of the CM scale makes it have theoretical concerns, and CM may measure the persistence of fear rather than that of courage (Howard and Alipour, 2014). The internal consistency coefficient of CM is 0.92, and the reliability of the retest after 3 weeks is 0.66. The medium level of retest reliability and the relatively small number of questions indicate some instability factors in the use of single-dimensional questionnaires to measure complex courage (Cheng and Huang, 2014).

Konter and Ng (2012) developed SCS suitable for teenagers aged 13–22 to fill the gap in the courage scale before. The scale has been widely used in other cultures and has versions suitable for different ages, with dependable reliability and validity. For example, Cronbach’s *α* of five dimensions of SCS (RSCS-C) for children aged 10–13 is MT = 0.75, DT = 0.76, AT = 0.71, VS = 0.75 and SB = 0.68, which has good structural validity (Konter et al., 2013). The revised version of the *Malaysian SCS* (SCS-M) keeps the same dimensions and adds two items based on the original scale. Cronbach’s *α* of the five dimensions is MT = 0.64, DT = 0.76, AT = 0.71, VS = 0.68, and SB = 0.69, which has acceptable structural validity (Hidrus et al., 2020). Konter et al. (2020b) revised the *SCS* for American college athletes (SCS-AE) and finally retained 24 items in four dimensions. Cronbach’s *α* in four dimensions is MT = 0.73, DT = 0.77, AT = 0.78, and VS = 0.74, which has good structural validity. Therefore, the psychological structure of sports courage may be influenced by culture and age.

Based on the above theoretical basis and practical requirements, it is difficult to directly apply the *SCS* to Chinese athletes. Therefore, the scale needs to be revised for Chinese athletes, and its reliability and validity shall be tested among college students to develop the *SCS* suitable for Chinese athletes.

## Materials and methods

### Participants

This study protocol has been approved by the Ethics Committee of Shenyang Sport University. A total of 775 college students were enrolled in the work, 683 of whom were included in the samples. The exclusion criteria are samples with incomplete basic information and missing questions. The age ranged from 15 to 24 years old, with an average age of 20.30 years old (*SD* = 2.50) in the valid questionnaire. There were 387 males (56.67%) and 296 females (43.33%). The average length of training was 5.26 years (*SD* = 2.59). Four hundred and seven people (59.59%) have achieved the skill level of athletes and 276 people (40.41%) have not. Sports mainly included skiing, skating, basketball, football, track and field, aerobics, table tennis, tennis, and badminton. Participants must sign an informed consent form.

### Measures

#### Sports courage scale

Konter and Ng (2012) developed the items of Sports courage scale (*SCS*) based on the experience and feeling of sports courage, and the items were generated according to the meaning of sports courage. The scale was divided into five dimensions: Mastery (MT), Determination (DT), Assertiveness (AT), Venturesome (VS), and Self-Sacrificial Behavior (SB). Mastery included 7 items; determination included 9 items; assertiveness included 7 items; venturesome included 4 items; self-sacrificial behavior included 4 items. They formed a total of 31 items (including 7 reverse scoring items; Konter and Ng, 2012). 5-point Likert scale was adopted for SCS, with 1 = “Totally disagree” and 5 = “Totally agree.” High scores indicated high sports courage.

#### Courage measure

Courage measure (CM) compiled by Norton and Weiss (2009) contains only one dimension, that is, persevering in moving forward despite experiencing fear. The questionnaire contains 12 question items, and the scores of each question item range from 1 = “Never” to 7 = “Always.” The higher the total score, the higher the level of courage.

#### Simple grit scale

Duckworth’s research team (Duckworth et al., 2007) revised and compiled grit scale (Grit-S). The scale is divided into two component tables including consistency of interest (CI) and persistence of effort (PE), with 8 items. The four items in the persistent effort dimension are scored in reverse. Likert’s 5-point scoring is used, 1 = “Completely inconsistent” and 5 = “Fully consistent.” A high score represents a high degree of perseverance.

### Procedure

The work was authorized by Dr. Erkut Konter to revise SCS. SCS was first translated into Chinese by 2 college English teachers and 1 graduate student in sports psychology in this scale, respectively. The translations of the above 3 people were integrated and compared, and an agreement was reached after discussion to form the first draft of the Chinese version of *SCS*. Then, the first draft of the Chinese version of *SCS* was retranslated into English by 1 college English teacher and 1 graduate student majoring in English without reading *SCS*. We compared the translated English with the original text, modified the items with great differences in translation, and further improved the accuracy of the questionnaire translation. Finally, two Chinese psychological professors and several psychological graduate students were asked to evaluate the validity of the content so that it could combine Chinese culture and semantics in terms of expression habits and life customs. This was to ensure that the Chinese version was consistent with the meaning expressed in the original scale so that it conformed to Chinese culture and semantics in terms of expression habits and living customs. Twenty-three Chinese athletes were randomly selected to complete the scale because they would understand. In the end, the final questionnaire was prepared.

Then the formal test was carried out with the questionnaire. First of all, on the front page of the electronic questionnaire, all athletes who took the test filled out an informed consent form. Secondly, athletes were asked to provide demographic data. Finally, they were asked to complete the questionnaire. The data collection process was undertaken by several graduate students in psychology. Questionnaires were collected by the principal investigators after the completion of the survey.

The investigators recruited 143 athletes offline in Shenyang Sport University, and they completed SCS twice in 3 weeks. These questionnaires were used to test the test–retest reliability of the scale.

### Data analysis

SPSS 23.0 and Mplus 8.0 were used to analyze the data. Project analysis was used to examine the degree of discrimination of projects. Internal consistency reliability coefficient and halved reliability coefficient were used for reliability analysis. Structural validity was tested by exploratory structural equation modeling (ESEM; Asparouhov and Muthén, 2009). Some common fitting indices were used to determine the fitting degree of the model, including Chi-square goodness-of-fit statistics, comparative fit index (CFI), Tucker-Lewis index (TLI), root mean square error of approximation (RMSEA), and standardized root mean square residual (SRMR).

## Results

### Project analysis

We initially calculate corrected item-total correlation *r* (Chen et al., 2015). *r* refers to the correlation coefficient between the scores of each item and the total score of the remaining items in each dimension. *r* of the items in MT is 0.326–0.788. *r* of items in DT is 0.202–0.425, and *r* of items 12 and 17 is 0.202 and 0.214, respectively. *r* of items in AT is 0.119–0.660, and *r* of items 3, 8, and 13 is 0.241, 0.119, and 0.224, respectively. *r* of items in VS is 0.622–0.777. *r* of items in SB is 0.073–0.627, and *r* of item 31 is 0.073. *r* of items 3, 8, 12, 13, 17, and 31 are less than 0.300, and *r* of all the other items is greater than 0.300 (see Table 1). Then, item-total correlation is calculated (Hao and Hong, 2014).

**Table 1:** Results of corrected item-total correlation *r*, independent sample *t*-test, and standardized factor load.

Item	Project content	Corrected *r* before deletion	Corrected *r* after deletion	*t* before deletion	*t* after deletion	Factor loading
1	遇到困难时, 我会害怕失败。(When faced with a difficult situation, I experienced a fear of failure.)	0.719^**^	0.719^**^	0.811^**^	0.811^**^	0.805
6	害怕使我很少获得比赛胜利。(I have limited success because I get frightened.)	0.788^**^	0.788^**^	0.857^**^	0.857^**^	0.859
11	在比赛中, 我经常会夸大困难。(I exaggerate difficulties.)	0.714^**^	0.714^**^	0.800^**^	0.800^**^	0.805
16	对自己能力的怀疑让我不能取得比赛胜利。(My doubts about my abilities keep me from success.)	0.715^**^	0.715^**^	0.797^**^	0.797^**^	0.800
21	缺乏自信让我错失了很多机会。(My lack of self-confidence makes me miss opportunities.)	0.710^**^	0.710^**^	0.792^**^	0.792^**^	0.790
24	当面临困难时, 我会变得很悲观。 (I become pessimistic when faced with difficulty.)	0.742^**^	0.742^**^	0.821^**^	0.821^**^	0.810
27	在困难的情况下, 我会选择逃避。(When I am in a difficult situation I take the easiest option.)	0.326^**^	0.326^**^	0.470^**^	0.470^**^	-
2	我不会逃避挑战强大的对手。(I do not avoid challenging strong opponents.)	0.367^**^	0.352^**^	0.549^**^	0.561^**^	0.853
7	我是一个相信任何事情都可以实现的人。 (I am a person that believes anything can be achieved.)	0.381^**^	0.372^**^	0.553**	0.569^**^	0.836
12	我会努力证明没有什么好害怕的。 (I struggle to demonstrate that there is nothing to fear.)	0.202^**^	-	0.411^**^	-	-
17	我能积极主动地面对困难。(I do not avoid taking the initiative when faced with difficult conditions.)	0.214^**^	-	0.402^**^	-	-
20	不管当前情况有多不利, 我也会尽我最大的能力。(I perform to the best of my ability not matter how negative the current conditions.)	0.376^**^	0.372^**^	0.547^**^	0.568^**^	0.834
22	我会竞争到最后而不害怕失败。 (I compete until the end without worrying about failure.)	0.361^**^	0.385^**^	0.549^**^	0.593^**^	-
25	我觉得我有实力在困难的条件下取得比赛胜利。(I feel that I have the strength to be successful in difficult conditions.)	0.347^**^	0.366^**^	0.531^**^	0.572^**^	-
28	即使遇到困难, 我也会很自信。(I am assertive even in difficult conditions.)	0.425^**^	0.452^**^	0.588^**^	0.631^**^	-
30	即使有压力, 我也不会忘记自己的目标。(Even when under pressure I do not lose sight of my goals.)	0.380^**^	0.401^**^	0.553^**^	0.593^**^	-
3	在比赛中, 我能轻松应对对手的进攻。 (It is easy for me to overcome my opponent’s attacks.)	0.241^**^	-	0.448^**^	-	-
8	我喜欢积极主动地面对困难。(I like to take initiative in the face of difficulties.)	0.119^**^	-	0.368^**^	-	-
13	在比赛中, 我能应对对手的突然进攻。 (I have no problems responding to opponents’ sudden attacks.)	0.224^**^	-	0.459^**^	-	-
18	我善于在遇到困难时寻找解决问题的办法。(I am good at finding solutions to problems in difficult conditions.)	0.660^**^	0.738^**^	0.789^**^	0.856^**^	0.837
23	即使面对危险, 我也会坚持自己完成比赛(任务)。(I assert myself even when facing hazards.)	0.602^**^	0.682^**^	0.740^**^	0.824^**^	0.813
26	即使面对危险, 我也会从容镇定地继续比赛。(I continue to compete without panicking even when faced with a danger.)	0.594^**^	0.731^**^	0.736^**^	0.855^**^	0.857
29	遇到困难时, 我不会推卸责任。(In difficult situations I do not shirk responsibility.)	0.576^**^	0.721^**^	0.721^**^	0.847^**^	0.852
4	为了不输掉比赛, 我会冒着受伤的风险 。(I risk injury in order not to lose.)	0.644^**^	0.644^**^	0.811^**^	0.811^**^	0.785
9	我甘愿冒任何受伤的风险以取得比赛胜利。(I would take any type of risks to become successful.)	0.642^**^	0.642^**^	0.806^**^	0.806^**^	0.763
14	即使面对危险的情况, 我也会去挑战。(I do not avoid a challenge even when facing a dangerous situation.)	0.777^**^	0.777^**^	0.879^**^	0.879^**^	0.884
19	即使面对受伤的可能, 我也会尽我最大的能力。(Even when facing the possibility of injury, I perform to the best of my ability.)	0.622^**^	0.622^**^	0.789^**^	0.789^**^	0.774
5	即使有失败的可能性, 我也会毫不犹豫地去参加比赛。(I do not hesitate to compete, even when facing the possibility of defeat.)	0.627^**^	0.681^**^	0.818^**^	0.862^**^	0.850
10	即使这个动作(练习)对我可能有害, 我也会坚定目标拼到最后。(I defend my beliefs until the end even if this action might prove harmful to me.)	0.537^**^	0.612^**^	0.777^**^	0.837^**^	0.761
15	我可以接受他人对我的原则或信念的批评。(I can take criticism of my principles or beliefs.)	0.570^**^	0.646^**^	0.779^**^	0.839^**^	0.851
31	即使失去的比得到的要多, 我也会去完成比赛。(I compete even if I have much more to lose than to gain.)	0.073^**^	-	0.421^**^	-	-

^**^p < 0.01; ^***^p < 0.001. Mastery: items 1, 6, 11, 16, 21, 24, and 27. Determination: items 2, 7, 12, 17, 20, 22, 25, 28, and 30. Assertiveness: items 3, 8, 13, 18, 23, 26, and 29. Venturesome: items 4, 9, 14, and 19. Self-sacrificial behavior: items 5, 10, 15, and 31.

Item-total correlation *r* refers to the correlation between the item and the total score of the corresponding dimension. The correlation coefficient between the total score of MT and the items in the dimension table is between 0.470 and 0.857, with *ps* < 0.01. The correlation coefficient between the total score of DT and the items in the dimension is between 0.402 and 0.588, with *ps* < 0.01. The correlation coefficient between the total score of AT and each item in this dimension is between 0.368 and 0.780, with *ps* < 0.01. The correlation coefficient between the total score of VS and each item in this dimension is between 0.789 and 0.879, with *ps* < 0.01. The correlation coefficient between the total score of SB and the items in this dimension is between 0.421 and 0.818, with *ps* < 0.01. All values are greater than 0.3. Finally, respondents were grouped according to the top and bottom 27% of the total score of each dimension. An independent sample *t*-test was used to compare the scores of the high and low groups of each item, and significant differences existed in all items (see Table 1).

The corrected item-total correlation, extreme grouping independent sample *t*-test, and item-total correlation are recalculated after deleting items 3, 8, 12, 13, 17, and 31. The corrected item-total correlation of DT, AT, and SB is 0.352–0.452, 0.682–0.738, and 0.612–0.681, respectively. The correlation coefficient between the total score of DT and the items in the dimension is between 0.561 and 0.631, with *p* < 0.01. The correlation coefficient between the total score of AT and each item is between 0.824 and 0.856, with *p* < 0.01. The correlation coefficient between the total score of SB and items is between 0.837 and 0.862, with *p* < 0.01. All the items in the scale are of good discrimination after deleting the above six items, so items 3, 8, 12, 13, 17, and 31 are considered to be deleted.

### Validity analysis

#### Exploratory structural equation modeling

A five-factor model of the original data was established after reverse-scoring related items. A first-order five-factor model is set for the original design theory of the questionnaire, including mastery (factor I), determination (factor II), assertiveness (factor III), venturesome (factor IV), and self-sacrificial behavior (factor V). Table 2 introduces the fitting index of the ESEM model. The fitting index shows that Model 1 (25 items) cannot fit data well. The factor load values of items 22, 25, 27, 28, and 30 are 0.062, 0.039, 0.346, 0.148, and 0.066 (Table 3). According to the correction of the model (Bagozzi and Heatherton, 1994; Liu, 2019), the topics of small load values can be deleted based on the load values of factors (threshold < 0.5). Therefore, items 22, 25, 27, 28, and 30 are to be deleted. The items in bold were retained.

**Table 2:** Goodness of fit and indices of different SCS models.

Model	*χ^2^*	*df*	*χ^2^/df*	CFI	TLI	RMSEA	SRMR
Model 1	1798.043^***^	265	6.785	0.805	0.779	0.092	0.099
Model 2	361.944^***^	160	2.262	0.969	0.963	0.043	0.044

^***^p < 0.001.

**Table 3:** Factor load values of model 1.

		Estimate	S.E.	Est./S.E.	Two-tailed *P*-value
MT	BY				
**1**		**0.769**	**0.018**	**43.009**	**0.000**
**6**		**0.852**	**0.013**	**64.568**	**0.000**
**11**		**0.771**	**0.018**	**43.274**	**0.000**
**16**		**0.760**	**0.018**	**41.335**	**0.000**
**21**		**0.757**	**0.019**	**40.808**	**0.000**
**24**		**0.788**	**0.017**	**46.739**	**0.000**
27		0.346	0.036	9.729	0.000
DT	BY				
**2**		**0.774**	**0.023**	**34.293**	**0.000**
**7**		**0.747**	**0.023**	**31.877**	**0.000**
**20**		**0.795**	**0.022**	**35.669**	**0.000**
22		0.062	0.043	1.446	0.148
25		0.039	0.043	0.916	0.360
28		0.148	0.042	3.491	0.000
30		0.066	0.043	1.523	0.128
VS	BY				
**4**		**0.717**	**0.022**	**32.065**	**0.000**
**9**		**0.732**	**0.022**	**33.847**	**0.000**
**14**		**0.881**	**0.016**	**56.162**	**0.000**
**19**		**0.693**	**0.023**	**29.614**	**0.000**
SB	BY				
**5**		**0.808**	**0.023**	**35.909**	**0.000**
**10**		**0.724**	**0.026**	**28.278**	**0.000**
**15**		**0.745**	**0.024**	**31.168**	**0.000**
AT	BY				
**18**		**0.820**	**0.017**	**47.187**	**0.000**
**23**		**0.744**	**0.021**	**35.824**	**0.000**
**26**		**0.800**	**0.018**	**44.079**	**0.000**
**29**		**0.787**	**0.019**	**42.070**	**0.000**

**Figure 1:**
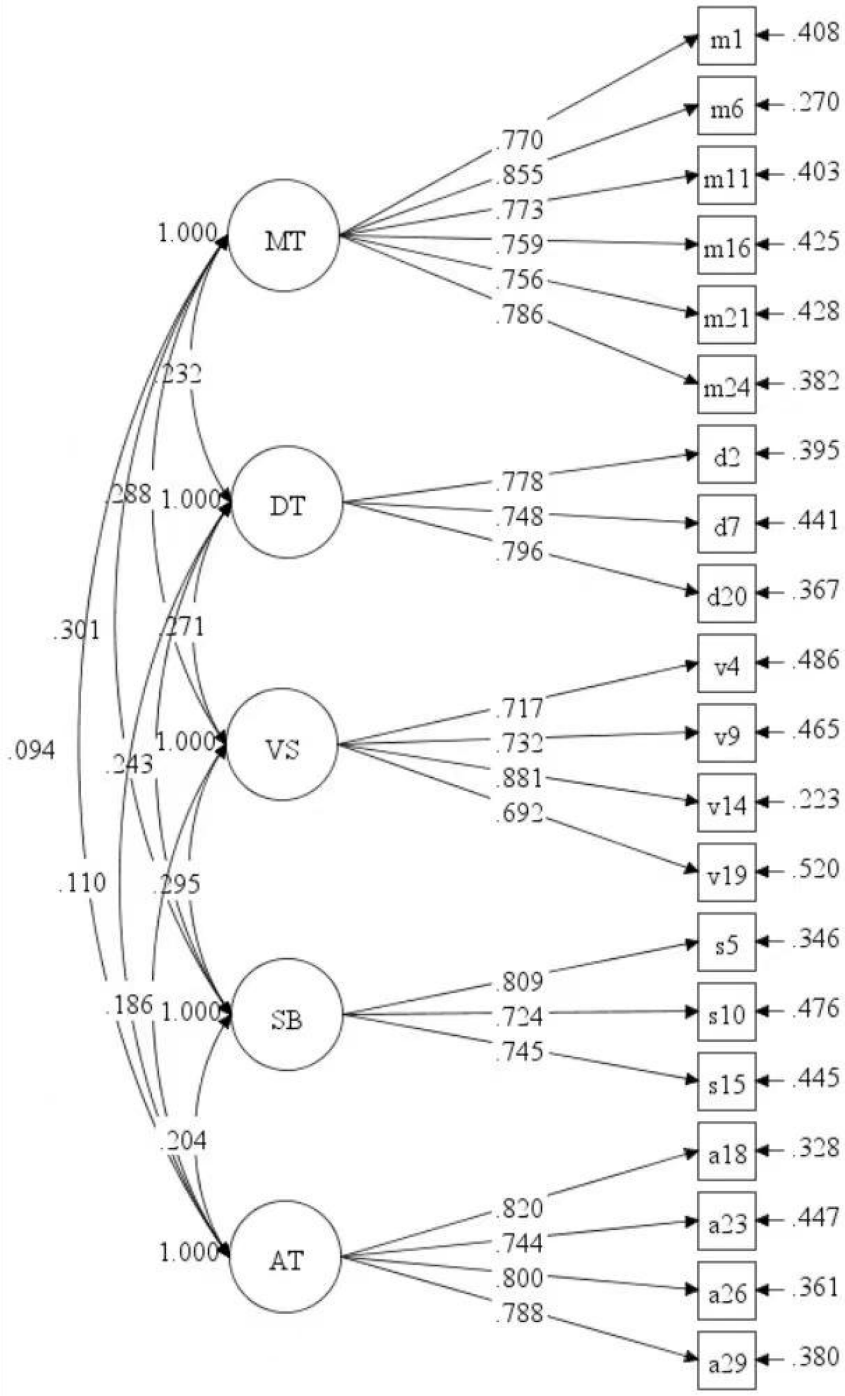
Model 2 with 20 items.

Verification factor analysis is performed again after deletion. Table 2 shows the fitting indices of model 2 (20 items): *χ^2^/df* is less than 3; CFI and TLI are greater than 0.90; RMSEA and SRMR are less than 0.05. These fitting indices all meet the requirements of psychometrics (Mai and Wen, 2013), indicating that the fitting indices of model 2 are more ideal (see Figure 1). Average variance extracted (AVE) of model 2 and the composite reliability (CR) are ideal (Fornell and Larcker, 1981; see Table 4 for the results).

**Table 4:** Model reliability and composite reliability.

Factor	AVE	CR
MT	0.614	0.905
DT	0.599	0.818
VS	0.576	0.844
SB	0.578	0.804
AT	0.621	0.868

#### Empirical validity

Empirical validity is also called predictive validity. Grouping was performed according to the presence or absence of exercise level. Then an independent sample *t*-test was used to compare the differences between the total scores of different grouping scales. The results showed that the total score of *SCS* in different groups was significantly different. *t* = 2.446 and *p* < 0.05, indicating that the scale has good empirical validity.

#### Criterion-related validity

CM and Grit-S were selected as the validity questionnaire, and criterion-related validity was tested on the overall sample. The results showed that the total score of SCS was positively correlated with the total scores of CM and Grit-S. Exercise courage was positively correlated with courage and perseverance (see Table 5 for the results).

**Table 5:** Correlation coefficients among SCS, CM, and Grit-S.

	1	2	3	4	5	6	7	8	9	10
1. MT	1									
2. DT	0.196^**^	1								
3. VS	0.254^**^	0.242^**^	1							
4. SB	0.270^**^	0.203^**^	0.267^**^	1						
5. AT	0.076^*^	0.089^*^	0.171^**^	0.171^**^	1					
6. SCS	0.744^**^	0.495^**^	0.636^**^	0.569^**^	0.470^**^	1				
7. CM	0.293^**^	0.236^**^	0.276^**^	0.233^**^	0.222^**^	0.426^**^	1			
8. CI	0.279^**^	0.259^**^	0.262^**^	0.289^**^	0.273^**^	0.448^**^	0.184^**^	1		
9.P E	0.238^**^	0.271^**^	0.294^**^	0.308^**^	0.264^**^	0.442^**^	0.135^**^	0.486^**^	1	
10. Grit-S	0.301^**^	0.307^**^	0.323^**^	0.346^**^	0.312^**^	0.517^**^	0.186^**^	0.865^**^	0.859^**^	1

^*^p < 0.05, ^**^p < 0.01.

#### Discriminant validity

The five dimensions of SCS were tested for discriminant validity. Heterotrait–monotrait ratio (HTMT) was used for testing in the work. The HTMT results between dimensions were all less than 0.85, indicating a distinction between all dimensions (Henseler et al., 2015; see Table 6 for the results).

**Table 6:** HTMT results.

	MT	DT	VS	SB	AT
MT	-				
DT	0.229	-			
VS	0.292	0.292	-		
SB	0.312	0.250	0.322	-	
AT	0.086	0.106	0.200	0.206	-

### Reliability analysis

Reliability analysis showed that Cronbach’s α of the five dimensions of SCS was MT = 0.905, DT = 0.817, AT = 0.867, VS = 0.839, and SB = 0.802. Cronbach’s*α* of the total scale was 0.845, and McDonald’s Omega ω of SCS is 0.868. The split-half reliability of the five dimensions was as follows: MT = 0.903, DT = 0.822, AT = 0.861, VS = 0.839, and SB = 0.801. The split-half reliability of the total scale was 0.920.

## Discussions

The work applied SCS to Chinese athletes to assess whether it could accurately assess the level of sports courage of Chinese athletes. The results showed that the reliability and validity of SCS (Chinese version) were satisfactory. SCS (Chinese version) contains 20 projects and five dimensions [Mastery (MT), Determination (DT), Assertiveness (AT), Venturesome (VS), and Self-Sacrificial Behavior (SB)]. The factor structure of SCS was confirmed by structural equation model. The item analysis shows that the remaining 20 items are of good item discrimination. The internal consistency reliability coefficients in each dimension of the scale are 0.905, 0.817, 0.867, 0.839, and 0.802, respectively, and the internal consistency reliability coefficient of the total scale is 0.845. The split-half reliability of each dimension is 0.903, 0.822, 0.861, 0.839, and 0.801, and that of the total scale is 0.920. The retest correlation of the scale is 0.948, which can meet the psychometric standard. Factor analysis shows that the internal structure of the revised questionnaire is consistent with that of the original one. The fitting indices of the corrected model show that χ^2^/*df* is less than 3; CFI and TFI are greater than 0.90; RMSEA and SRMR are less than 0.08. Therefore, all fitting indices meet the psychometric standards, and the scale shows a clear structure.

Compared with the original version of the courage scale, the dimensions of SCS for Chinese athletes were consistent with those of the original scale (Konter and Ng, 2012). However, only 20 items in the original scale were retained, and these deleted items might be deleted due to the differences between China and Turkey. Group differences appeared for Chinese athletes during filling out questionnaires because of the influence of different countries in China and Turkey on the differences in athlete training models. Since the questionnaire for the work was only distributed in the north of China, regional differences in China had certain influences. Besides, the proportion of subjects might be a reason. There were 407 subjects with exercise levels in the work, which accounted for 59.59%. However, professional athletes accounted for less than 10% of the subjects of the initial version of SCS.

An interesting finding is that significant gender differences are found in MT. Male athletes score higher than female athletes in MT and SCS. The findings are consistent with the results of previous studies (Konter, 2016). Another previous study found that women’s emotions are stronger, last longer, and express more clearly; while men are more rational and can better control their emotions (Brody and Hall, 2008). There is a highly positive correlation degree between exercise level and exercise courage. Therefore, we should pay attention to how to improve courage through exercise in future research. Significant differences exist in sports courage between athletes in winter sports and summer sports. A previous study found that compared with cross-country skiers, marathon runners look smarter and tougher on 16 Personality Factor Inventory (16 PF). They are more subjective, creative, and academic on derivative factors (Jerome and Valliant, 1983). Therefore, in future research, we should pay attention to the personality differences between athletes in summer and winter.

*Grit-S* and *CM* were used as questionnaires to test the criterion-related validity of *SCS*. The results showed a significant positive correlation between SC and perseverance. Perseverance refers to passion and persistence in long-term goals (Duckworth et al., 2007). Perseverance can be understood as promoting sports professional skills by extending time to participate in and adhere to practical activities (Hodges et al., 2017). Schimschal et al. (2022) established a psychological-resource model of perseverance. Interest is defined as a psychological resource of perseverance, which enables a person to explore and deepen their interests through attributes such as curiosity, self-awareness, courage, and patience. A study by Howard and Cogswell (2019) also showed that behavioral social courage is positively correlated with perseverance. Besides, the results show that the five dimensions of *SCS* are significantly positively correlated with courage.

The revised version of *SCS* for college athletes combined with American college students only retains 24 items in four dimensions of the original scale (Konter et al., 2020b). In this regard, it is reasonable to delete the items in the revised version of SCS for Chinese athletes. Gauvin et al. (1993) found that there may be serious misinterpretations and errors in the test. Besides obvious language differences, even if the test content is accurately translated, subtler cultural differences affect the test results.

In conclusion, the revised version of SCS is of good reliability and validity and can be used as a tool to measure the sports courage of Chinese athletes.

## Limitations and future directions

There are limitations in this study. (1) A self-reporting method is used, so these results may be affected by the effect of social identity. However, the work aims to develop a reliable and effective tool. (2) Athletes are selected as participants, but non-clinical samples are used. Clinical samples will be selected from athletes to expand the applicability of the scale. (3) Samples are relatively small and may not represent all Chinese athletes. Larger samples are needed for further evaluation in the future.

The findings have some practical significance. The work has taken the first step in applying SCS to Chinese. Future studies will use a wider range of athletes as participants, and it is recommended to check their reliability and effectiveness. Besides, there are significant differences in sports courage between athletes in summer and winter as well as between sports levels and non-sports levels. Athletic courage can be cultivated from exercise methods to enhance overall courage, which has a profound impact on individual development.

## Data availability statement

The original contributions presented in the study are included in the article/Supplementary material, further inquiries can be directed to the corresponding author.

## Author contributions

SG reviewed the literature and wrote the manuscript. SG, ZG, and RZ analyzed and dealt with the data. SG, RZ, and JJ Collected questionnaires. All authors contributed to the article and approved the submitted version.

## Funding

This work was supported by the Scientifific Research Fund Project of Education Department of Liaoning Province (WQN2020ST09).

## Conflict of interest

The authors declare that the research was conducted in the absence of any commercial or financial relationships that could be construed as a potential conflict of interest.

## Publisher’s note

All claims expressed in this article are solely those of the authors and do not necessarily represent those of their affiliated organizations, or those of the publisher, the editors and the reviewers. Any product that may be evaluated in this article, or claim that may be made by its manufacturer, is not guaranteed or endorsed by the publisher.
